# Supplementary figures and images for: Evidence from the resurrected family Polyrhabdinidae Kamm, 1922 (Apicomplexa: Gregarinomorpha) supports the epimerite, an attachment organelle, as a major eugregarine innovation
Source: PeerJ. 2021 Sep 16;9:e11912. doi: 10.7717/peerj.11912 (PMC8450007; doi:10.7717/peerj.11912)

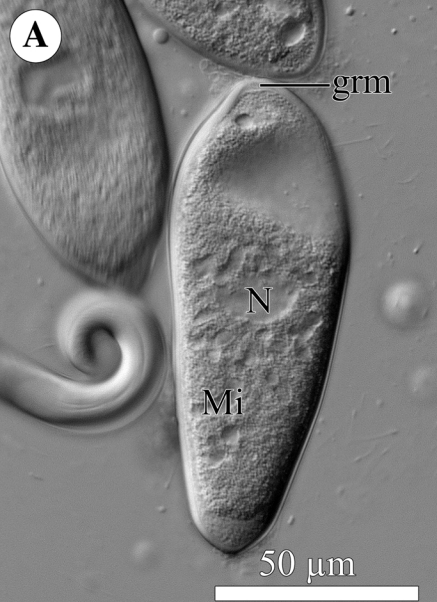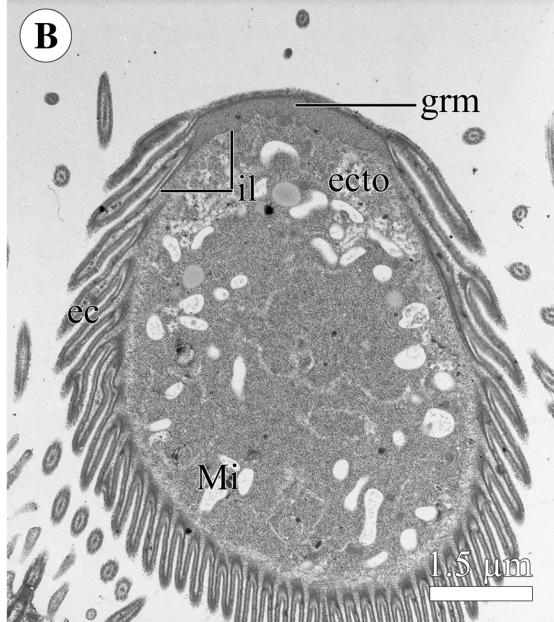

Supplement: Supplemental Information 1 — All micrographs show gamonts infected with metchnikovellid microsporidia (Mi, presporogonial stage development). (A) Slightly compressed, detached gamont without the epimerite. Note the granular material (grm) in the ectoplasm of the anterior end and the nucleus (N). Differential interference contrast. (B) Oblique longitudinal section through the anterior end of a detached gamont without the epimerite. Note the granular material (grm) in the ectoplasm (ecto) of the anterior end, epicytic crests (ec), the internal lamina (il), and the nucleus (N). [file peerj-09-11912-s001.pdf]

Ancoroidea

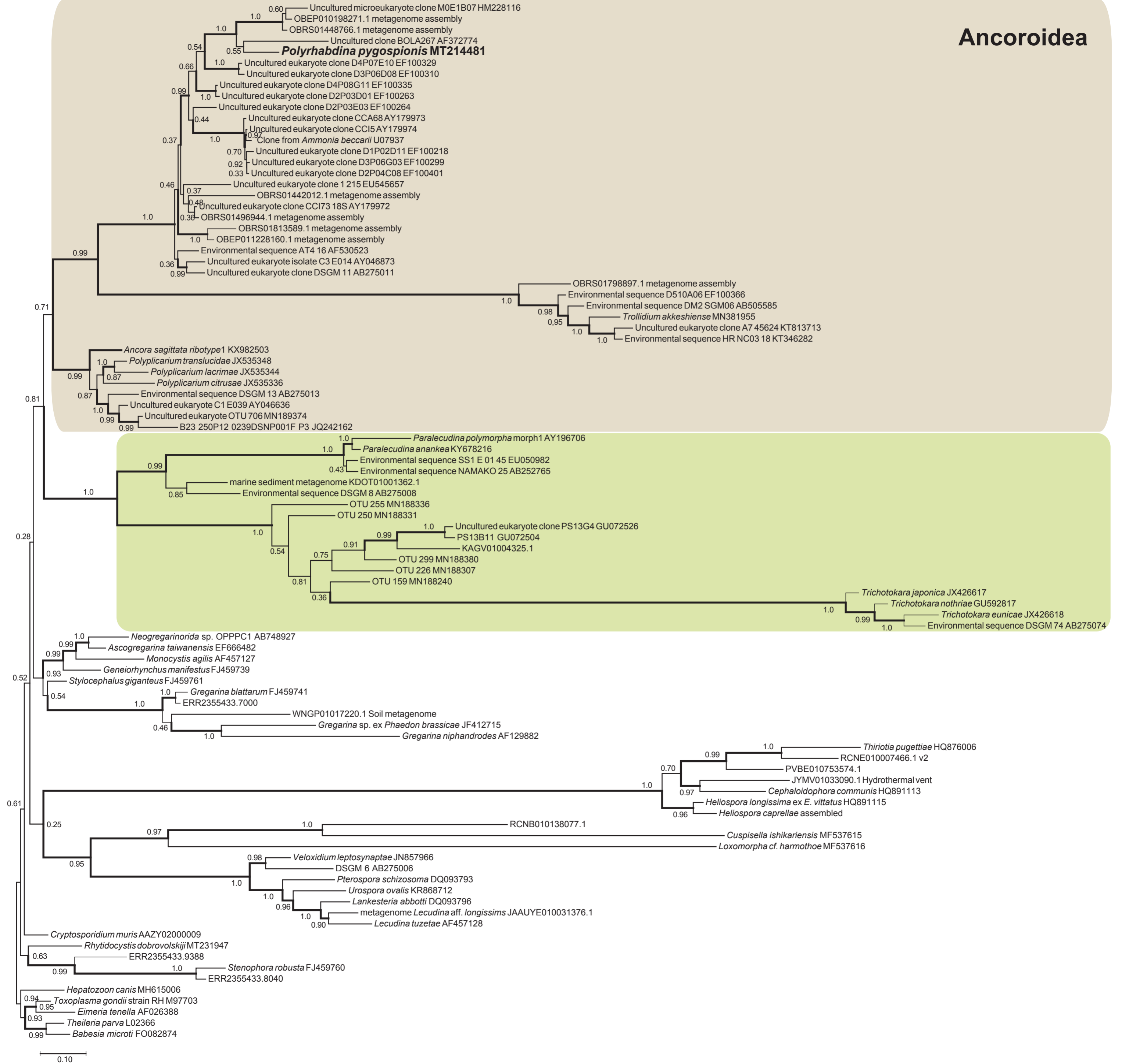

Supplement: Supplemental Information 2 — Numbers at branches indicate Bayesian posterior probabilities. The newly obtained sequence of Polyrhabdina pygospionis is in bold. [file peerj-09-11912-s002.pdf]

FULL

1471

1366

1257

1126

1087

828

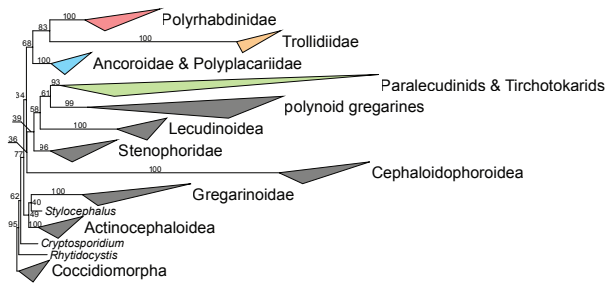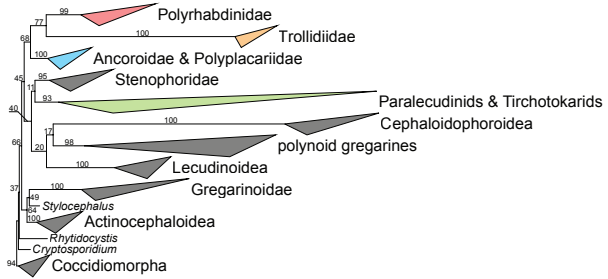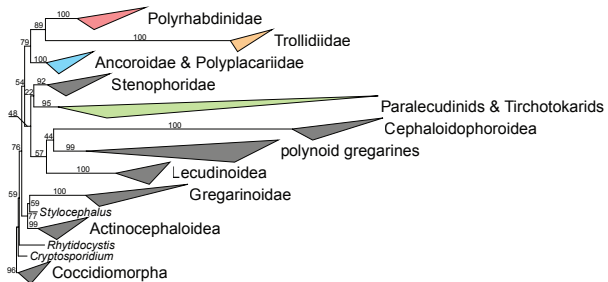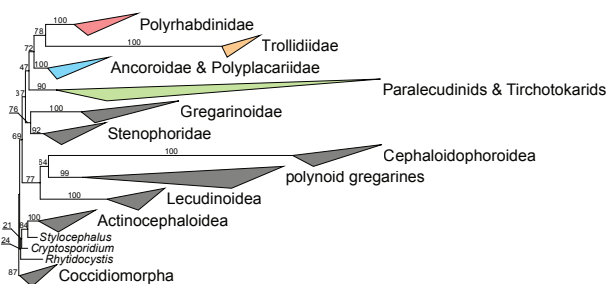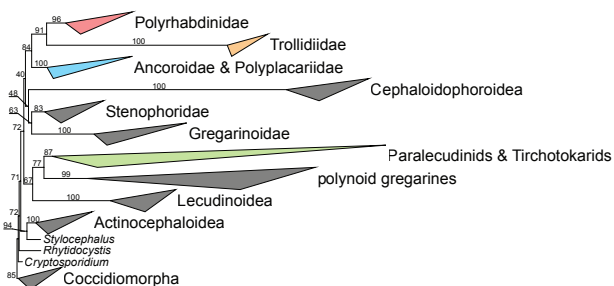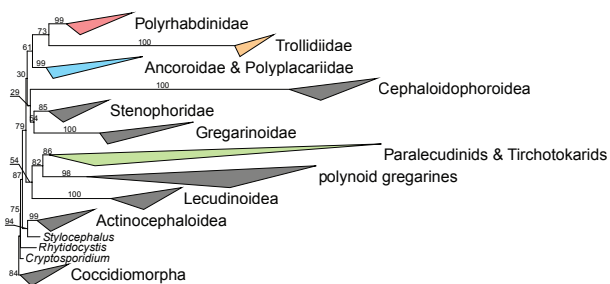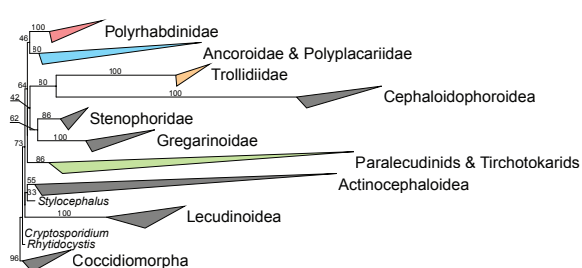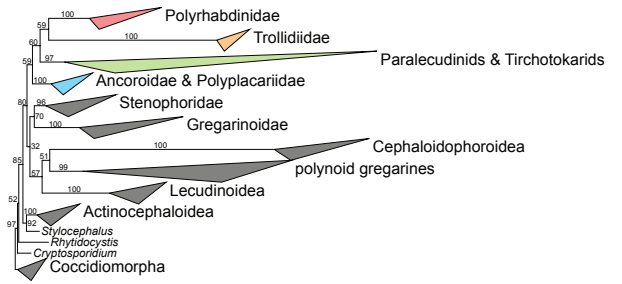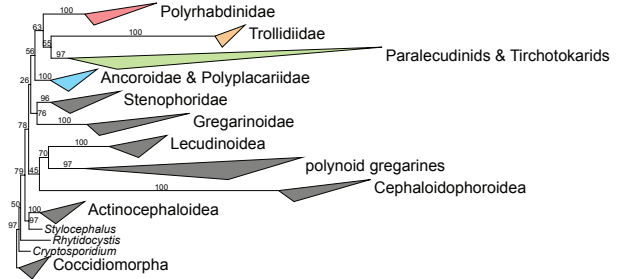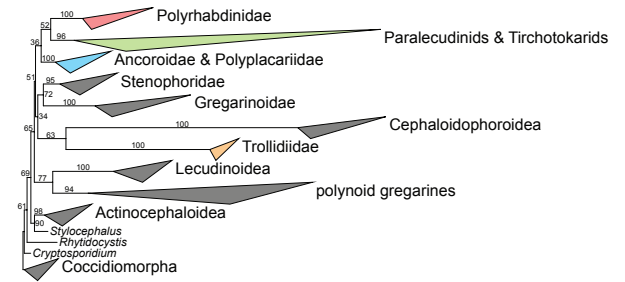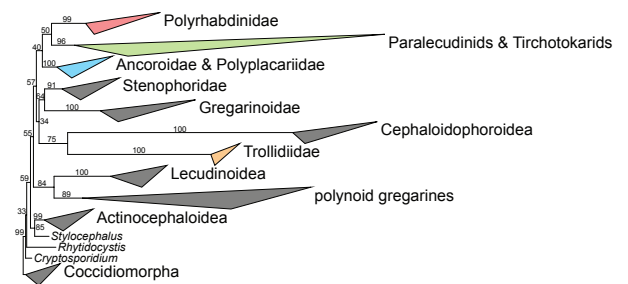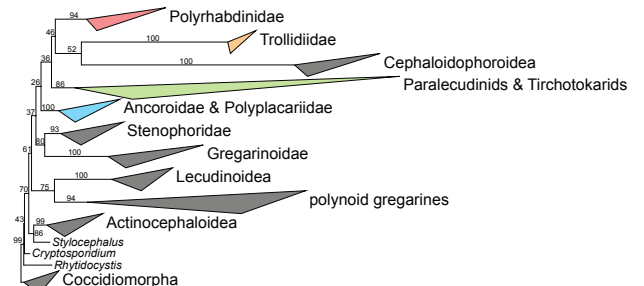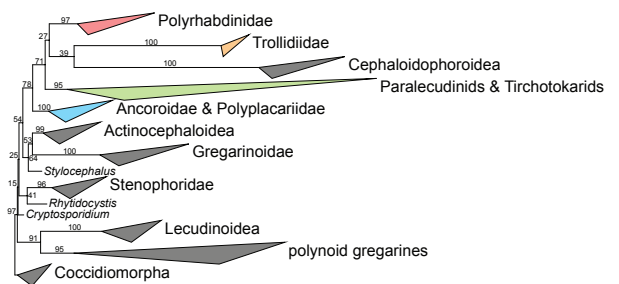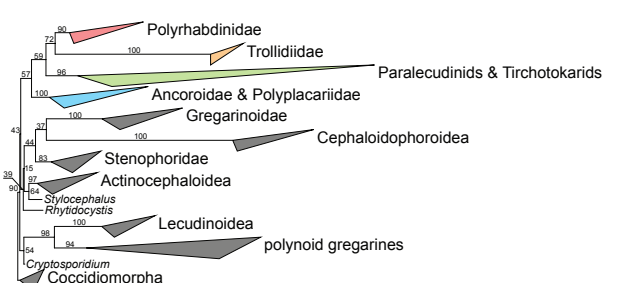

Supplement: Supplemental Information 3 — Maximum likelihood trees recovered from 1,578, 1,471, 1,366, 1,257, 1,126, and 828-site MAFFT E-INS-i + X-INS-i + trimAl and 1,574, 1,471, 1,366, 1,257, 1,126, and 828-site MAFFT E-INS-i + GUIDANCE2 alignments under GTR+F+I+G8 model with 1000 UFBoot replicates using IQ-TREE 2.1.2 (Minh et al., 2020). Numbers at branches indicate bootstrap (UFBoot) percentage supports. [file peerj-09-11912-s003.pdf]

# Ancoroidea

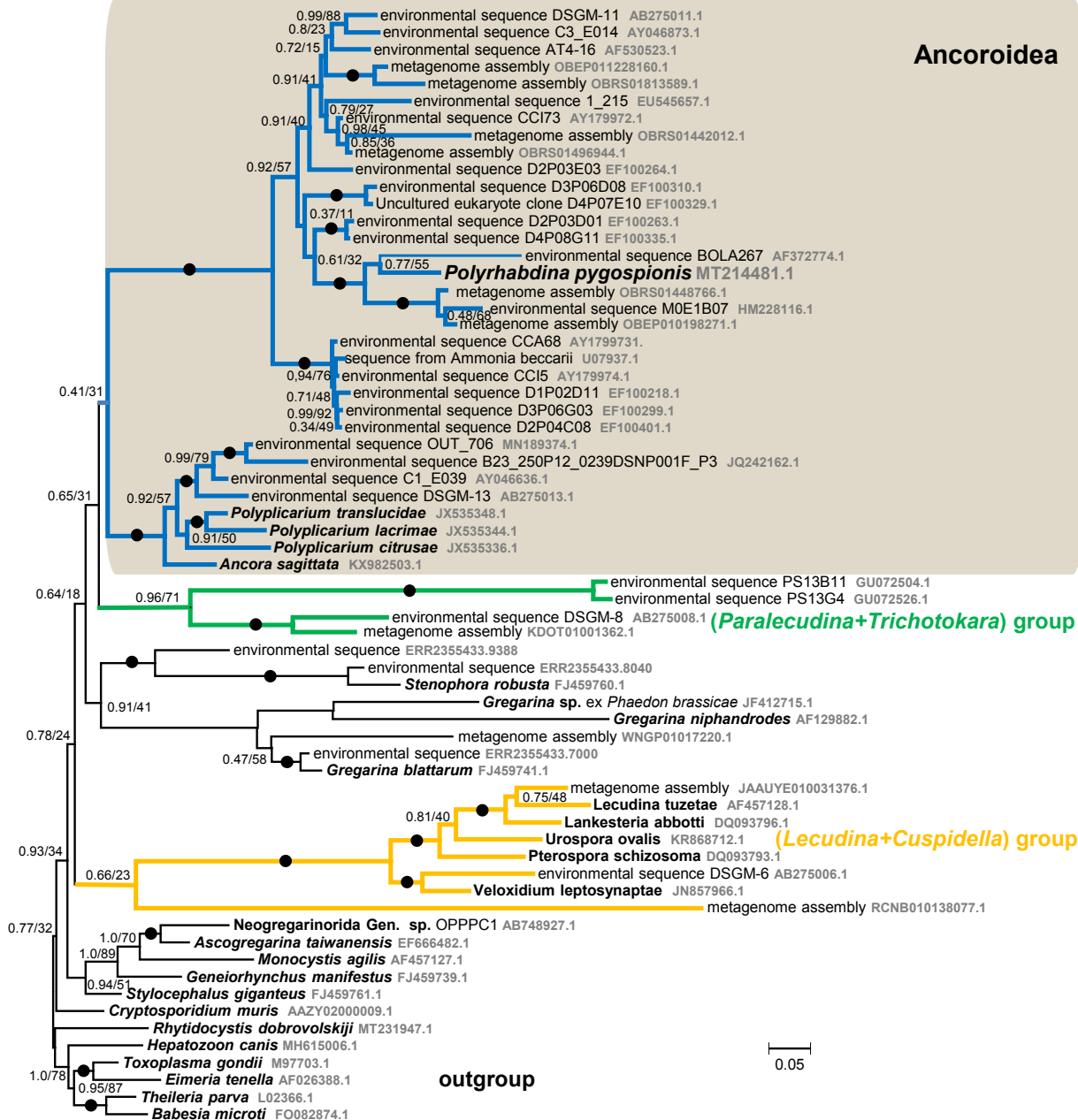

Supplement: Supplemental Information 4 — Numbers at branches indicate Bayesian posterior probabilities (numerator) and ML bootstrap percentage (denominator). Black dots on the branches indicate Bayesian posterior probabilities and bootstrap percentages of 1.0 and 95% and higher, respectively. The newly obtained sequence of Polyrhabdina pygospionis is in bold. [file peerj-09-11912-s004.pdf]

A

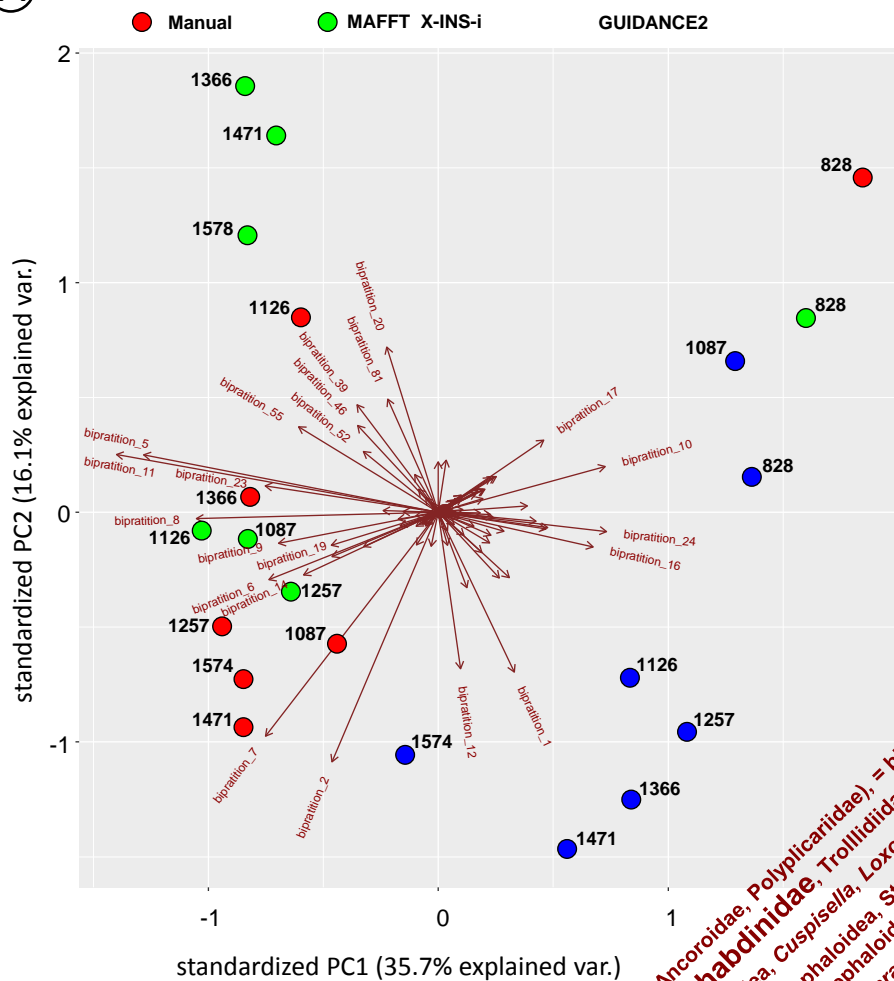

B

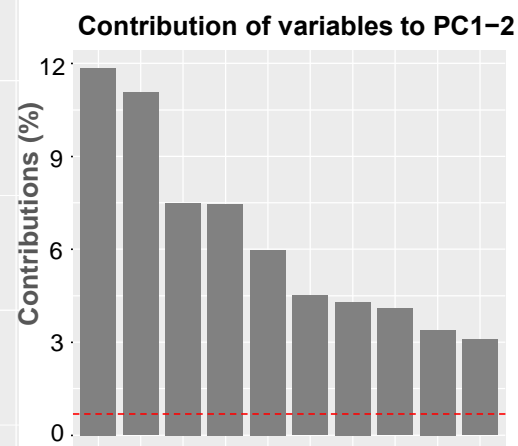

C

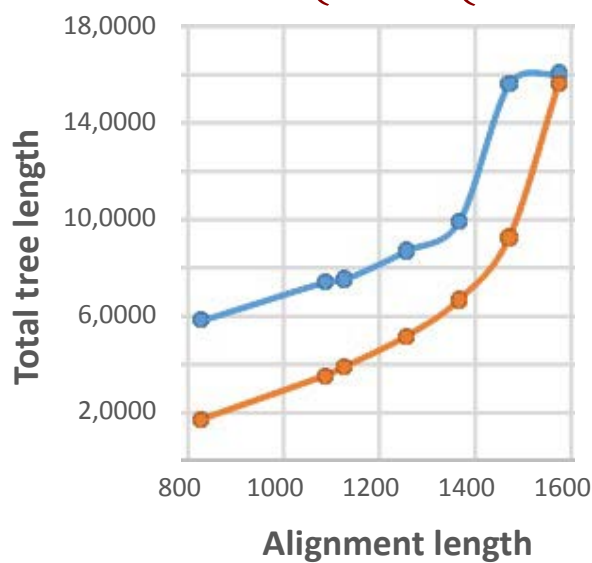

D

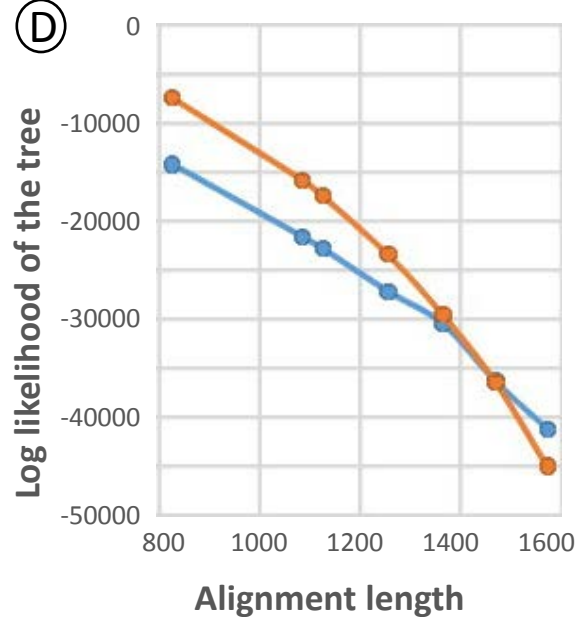

Supplement: Supplemental Information 5 — (A) Principal component analysis of alignments based on bipartition support values obtained in the ML analyses with UFBoot; twenty bipartitions with the most contributions to the principal components 1 and 2 are shown. Note that red dots formed a denser group than green or blues ones. Dot 828 indicates a critical level of data reduction at which the resolution of the trees is minimized. (B) Histogram of contribution values in percentages for the first ten bipartitions shown in A. (C–D) Comparison of two from the three alignments sets (Manual edited and GUIDANCE2; MAFT- X-INS-i is simalr to manual edited) in total tree length (C) and log likelihoods of trees (D); for both graphs, the maximum likelihood trees were used. [file peerj-09-11912-s005.pdf]

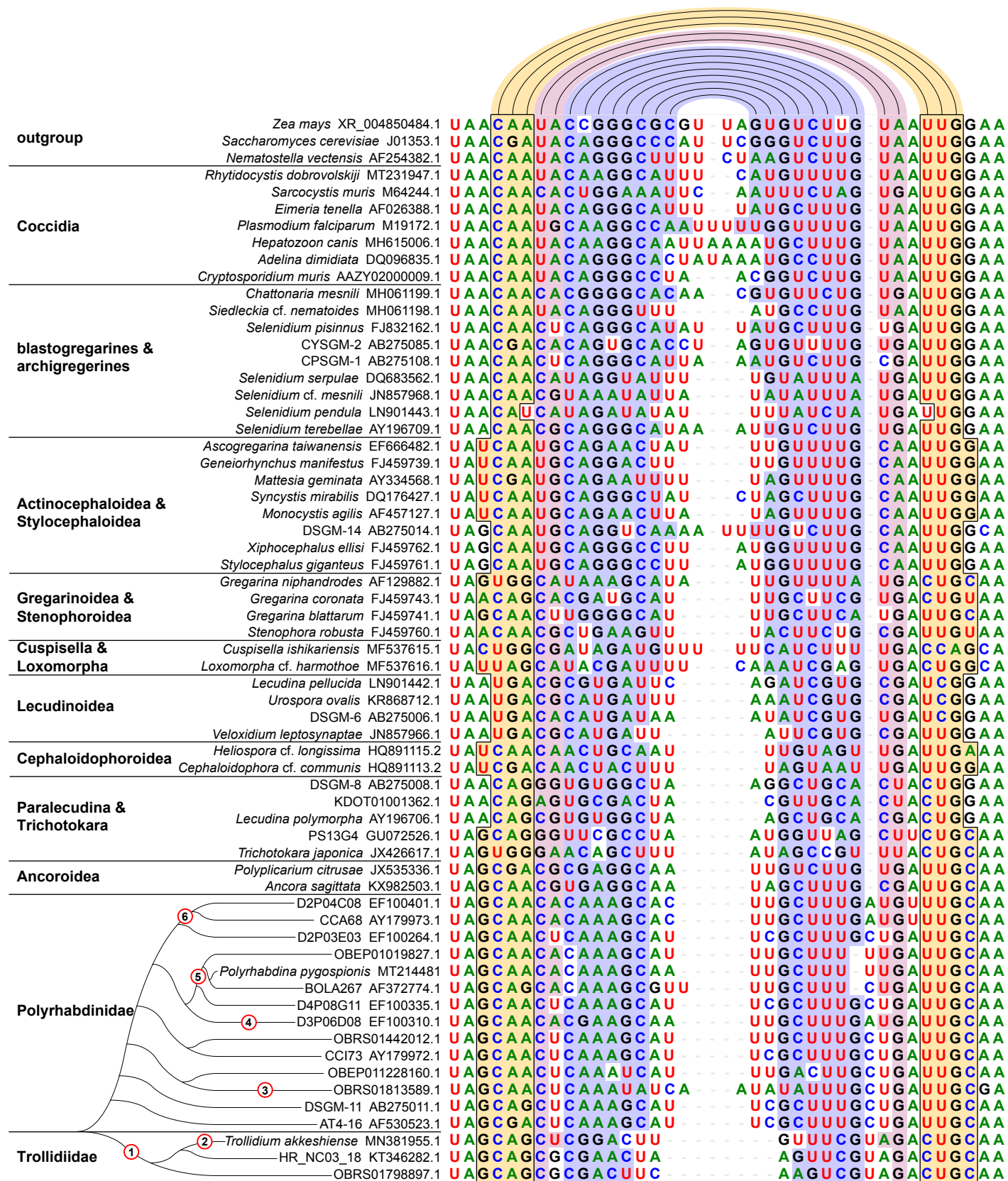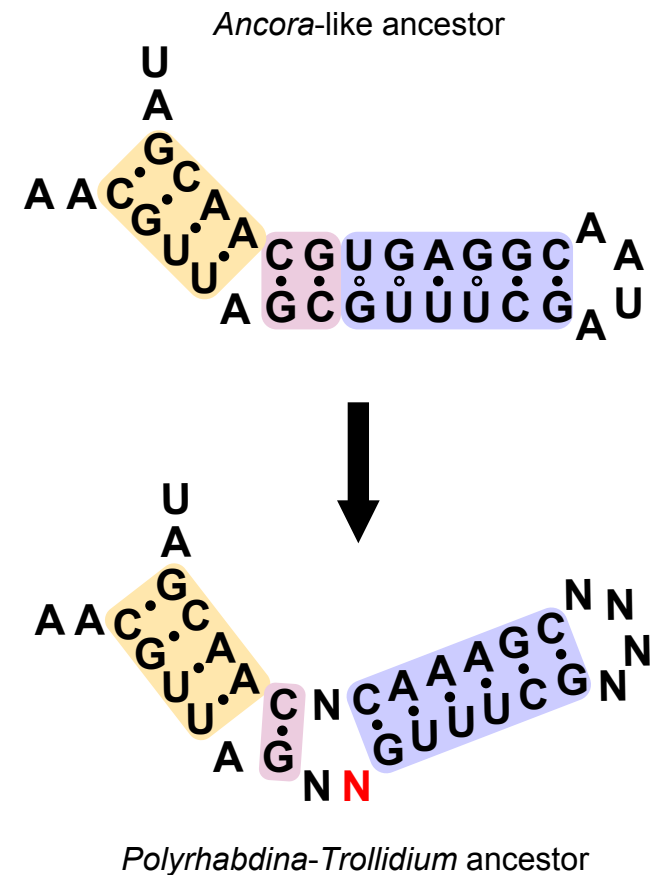

Supplement: Supplemental Information 6 — Complementary nucleotides of the helices are shaded; the proposed evolutionary transition marked by a single nucleotide insertion and uniting the families Polyrhabdinidae and Trollidiidae is depicted schematically on the right; the corresponding scenario for the evolution of the helix 17 region within families Polyrhabdinidae and Trollidiidae is outlined in the tree (lower left) with at least 6 transitions: 1–deletion of 1 bp from the helix; 2 –expansion of the loop by 1 bp into the helix; 3 –1bp insertion in a single OTU; 4 –transformation of the inner loop into a bulge; 5 –one nucleotide deletion in the apical part of the 3′-strand (resulting in an internal loop –2 bp –bulge); 6 –transformation of the inner loop into a bulge (resulting in a bulge –2 bp –a bulge). The names of major eugregarine lineages correspond to (Simdyanov et al., 2017; Cavalier-Smith, 2014). [file peerj-09-11912-s006.pdf]
