# Supplementary material for: Evidence from the resurrected family Polyrhabdinidae Kamm, 1922 (Apicomplexa: Gregarinomorpha) supports the epimerite, an attachment organelle, as a major eugregarine innovation
Source: PeerJ. 2021 Sep 16;9:e11912. doi: 10.7717/peerj.11912 (PMC8450007; doi:10.7717/peerj.11912)
Supplement: Supplemental Information 8 — Abbreviations: av, average; SD, standard deviation; n, number of measurements. [file peerj-09-11912-s008.docx]

**Supplement Material STable 1. Morphometry of investigated eugregarines.**

| **Species /**  **measurements** | ***Polyrhabdina pygospionis*** | ***Polyrhabdina* cf. *spionis*** |
| --- | --- | --- |
| Gamont, length x width, µm (av [SD], n) | 28–288.4 x 14–50.4 (123.3 [47.7] x 34.8 [6.8], 40) | 30 - 64.4 x 10.5 to 22.3 (53.4 [8.9] x 16.8 [2.7], 26) |
| Nucleus of gamonts, length x width, µm (av [SD], n) | 9.5–19.0 x 9.5–17.0 (14.8 [2.3] x 13.9 [1.8], 19) | -- |
| Nucleolus in large gamonts, maximal diameter, µm (av [SD], n) | 3.7–8.5 (6.2 [1.1], 17) | -- |
| Nucleoli in small gamonts, maximal diameter, µm (av [SD], n) | 2.7–5.9 (4.3 [1.1], 13) | -- |
| Density of crests, min x max per 1 µm (av, n) | 4 – 5 (5, 5) | 2–6 (4, 15) |
| Crests, length x width, µm (av [SD], n) | 0.9-2.4 x 0.2-0.5 (1.2 [0.5] x 0.3 [0.1], 19) | -- |
| Amylopectin granules, maximal diameter, µm (av [SD], n) | 0.1-1.3 (0.8 [0.3], 30) | -- |
| Thickness of the ectoplasm, µm (av. [SD], n) | 0.2 to 0.6 (0.4 [0.4], 14) | -- |
| Epimerite, length, µm (av, n) | 0.5 - 7.3 (3.9, 2) | -- |
| Epimerite, base diameter, µm (av, n) | 0.7 - 9.6 (5.0, 2) | 3.7–4.9 (4.3, 10) |
| Epimerite collar, length, µm (av, n) | 0.1 - 2.8 (1.6, 8) | 0.2–1.7 (1.2, 4) |
