# Supplementary material for: Evidence from the resurrected family Polyrhabdinidae Kamm, 1922 (Apicomplexa: Gregarinomorpha) supports the epimerite, an attachment organelle, as a major eugregarine innovation
Source: PeerJ. 2021 Sep 16;9:e11912. doi: 10.7717/peerj.11912 (PMC8450007; doi:10.7717/peerj.11912)
Supplement: Supplemental Information 9 — Anc –Ancoridae and Polyplicariidae, Poly –Polyrhabdinidae, Troll –Trollidiidae, Trich, Paralec –Trichotokara, Paralecudina and related environmental sequences, Ceph –Cephaloidiphoroidea, c-ELW –Expected Likelihood Weight (Strimmer and Rambaut 2002), p-AU –p-value of approximately unbiased (AU) test (Shimodaira, 2002). Plus signs denote the 95% confidence sets. Minus signs denote significant exclusion. All tests performed 10,000 resamplings using the RELL method in IQ-TREE 2.1.2 (Minh et al., 2020). [file peerj-09-11912-s009.docx]

**Supplement Material STable 2. Testing of possible compositions for Ancoroidea.**

| Alignment | Constrained topology | c-ELW | p-AU |
| --- | --- | --- | --- |
| GUIDANCE2 1574 bp | ((Anc,(Poly,Troll)),(others)); | 0.24 + | 0.446 + |
|  | ((Anc,(Poly,Trich,Paralec)),(others)); | 0.174 + | 0.353 + |
|  | ((Anc,(Poly,Troll,Trich,Paralec)),(others)); | 0.333 + | 0.6 + |
|  | ((Anc,(Poly,Troll,Trich,Paralec,Ceph)),(others)); | 0.252 + | 0.476 + |
| GUIDANCE2 1471 bp | ((Anc,(Poly,Troll)),(others)); | 0.282 + | 0.438 + |
|  | ((Anc,(Poly,Trich,Paralec)),(others)); | 0.19 + | 0.433 + |
|  | ((Anc,(Poly,Troll,Trich,Paralec)),(others)); | 0.41 + | 0.632 + |
|  | ((Anc,(Poly,Troll,Trich,Paralec,Ceph)),(others)); | 0.118 + | 0.323 + |
| xinsi 1578 bp | ((Anc,(Poly,Troll)),(others)); | 0.635 + | 0.696 + |
|  | ((Anc,(Poly,Trich,Paralec)),(others)); | 0.0287 - | 0.0735 + |
|  | ((Anc,(Poly,Troll,Trich,Paralec)),(others)); | 0.261 + | 0.48 + |
|  | ((Anc,(Poly,Troll,Trich,Paralec,Ceph)),(others)); | 0.0753 + | 0.224 + |
| xinsi 1471 bp | ((Anc,(Poly,Troll)),(others)); | 0.59 + | 0.711 + |
|  | ((Anc,(Poly,Trich,Paralec)),(others)); | 0.0313 - | 0.0663 + |
|  | ((Anc,(Poly,Troll,Trich,Paralec)),(others)); | 0.232 + | 0.481 + |
|  | ((Anc,(Poly,Troll,Trich,Paralec,Ceph)),(others)); | 0.147 + | 0.28 + |

Anc – Ancoridae and Polyplicariidae, Poly – Polyrhabdinidae, Troll – Trollidiidae, Trich, Paralec – *Trichotokara*, *Paralecudina* and related environmental sequences, Ceph – Cephaloidophoroidea, c-ELW – Expected Likelihood Weight (Strimmer & Rambaut 2002), p-AU – p-value of approximately unbiased (AU) test (Shimodaira, 2002). Plus signs denote the 95% confidence sets. Minus signs denote significant exclusion. All tests performed 10000 resamplings using the RELL method in IQ-TREE 2.1.2 (Minh et al., 2020).
