# Supplementary material for: Evidence from the resurrected family Polyrhabdinidae Kamm, 1922 (Apicomplexa: Gregarinomorpha) supports the epimerite, an attachment organelle, as a major eugregarine innovation
Source: PeerJ. 2021 Sep 16;9:e11912. doi: 10.7717/peerj.11912 (PMC8450007; doi:10.7717/peerj.11912)
Supplement: Supplemental Information 10 — Abbreviations: ‘—‘, no data; ‘?’, contradictory or vague description; ‘*’, species examined by electron scanning microscopy; ‘**’, species examined by electron scanning and transmission microscopy. The validation of the scientific names was conducted in the World Register of Marine Species (WoRMS). [file peerj-09-11912-s010.doc]

**Supplementary Material STable 3. Diagnostic characters of eugregarines of the genus *Polyrhabdina* Mingazinni, 1891.**

| **Species** | *Polyrhabdina spionis* (von Kölliker, 1845) Mingazzini, 1891, **type species** | *Polyrhabdina bifurcata* (Mackinnon, Ray, 1931) Reichenow, 1932 | ***Polyrhabdina cf. P. spionis** (von Kölliker, 1845) Mingazzini, 1891** | *Polyrhabdina brasili* Caullery et Mesnil, 1914 | *Polyrhabdina madrynense** Rueckert et al., 2018 | *Polyrhabdina minuta* Ganapati, 1946 | *Polyrhabdina polydorae** (Léger, 1893) Caullery et Mesnil, 1914 | *Polyrhabdina sp.* | *Polyrhabdina pygospionis* Caullery, Mesnil, 1914 | ***Polyrhabdina pygospionis*** Caullery, Mesnil, 1914, redescription** |
| --- | --- | --- | --- | --- | --- | --- | --- | --- | --- | --- |
| **Synonyms** | *Gregarina spionis* von Kölliker, 1845 | *Gregarina spionis* von Kölliker, 1845; *Polyrhabdina spionis* var. *bifurcatа* Mackinnon, Ray, 1931 |  |  |  |  | *Doliocystis polydorae* Léger, 1893; *Lecudina polydorae* Kamm, 1922 | *Doliocystis* sp. | *Polyrhabdina pygospionis* Caullery, Mesnil, 1914 emend. Paskerova et al. | |
|  | synonyms (?), *Polyrhabdina spionis* (von Kölliker, 1845) Mingazzini, 1891 | | |  |  |  |  |  |  |  |
| **Host name(s) (synonyms)** | *Malacoceros fuliginosus* (Claparède, 1870) (*Scolelepis fuliginosa* (Claparède, 1868), *Spio fuliginosus* Claparède, 1868) (M. 1891; C.& M. 1914; R. 1932)(?) *Malacoceros tetracerus* (Schmarda, 1861) (*Scolelepis ciliata* (Keferstein, 1862)) (C. & M. 1914) | *Malacoceros fuliginosus* (Claparède, 1870) (*Scolelepis fuliginosa* (Claparède, 1868)) | ***Malacoceros fuliginosus* (Claparède, 1870) (*Scolelepis fuliginosa* (Claparède, 1868))** | *Spio martinensis* Mesnil, 1896 | *Spio quadrisetosa* Blake, 1983 | *Prionospio cirrifera* Wirén, 1883 | *Polydora ciliata* (Johnston, 1838) (*Polydora agassizii* Claparède, 1869)*Dipolydora flava* (Claparède, 1870) (*Polydora flava* Claparède, 1870) (M. & R. 1931) | *Dipolydora socialis* (Schmarda, 1861) (*Polydora socialis* Schmarda, 1861) | *Pygospio elegans* Claparède, 1863 (*P. seticornis* in Caullery and Mesnil, 1914a,b, 1919) | ***Pygospio elegans* Claparède, 1863** |
| **Type locality** | Gulf of Naples, Mediterranean Sea | Plymouth, English Channel, North East Atlantic | **Roscoff, English Channel, North East Atlantic** | Cap de la Hague, English Channel, North East Atlantic | Puerto Madryn, Argentina, South West Atlantic | Chennai (Madras), Bay of Bengal, Indian Ocean | Gulf of Marseilles, Mediterranean Sea (L. 1893);  Plymouth, English Channel, North East Atlantic (M. & R., 1931 for *Dipolydora flava*) | Rio de Janeiro, Brazil, South West Atlantic | Anse Saint-Martin, English Channel, North East Atlantic | **Kandalaksha Bay, White Sea** |
| **Localization in the host** | intestine | intestine | **intestine** | intestine | intestine | hindgut | Intestine | intestine | intestine | **intestine** |
| **Infection:** [**extensiveness**](http://www.lingvo-online.ru/ru/Search/Translate/GlossaryItemExtraInfo?text=экстенсивность&translation=extensiveness&srcLang=ru&destLang=en)**, intensity** | sometimes heavy infection (in summer) (M. 1891) | 85 (34%) out of 250 polychaetes, sometimes heavy (M. & R. 1931) | **3 (17%) out of 18 polychaetes, sometimes heavy** | -- | -- | low extensiveness & intensity | 75% of polychaetes, sometimes heavy (M. & R. 1931) | -- | --  generally abundant (C.et M., 1919) | **abundant; about 42%, 1-50 per host** |
| **Trophozoite shape** | spindle-shaped (von K. 1848); elliptical, pear-shaped (M. 1981); young trophozoites conical; gamonts oval, slightly flattened (C. & M. 1914) | sac-like, circular in cross section (M. & R. 1931) | **spindle-, rhomboid-shaped** | ellipsoid (fig. C 1-3, E1 in C.et M. 1919) | rhomboid or ellipsoid | young trophozoites bottle-shaped with well-developed epimerite; gamonts elliptical, with short neck at the anterior end | sac-like (M. & R. 1931); oval to ellipsoidal (R. et al. 2018) | --  ellipsoid (fig. 6-7, de F. et al. 1918) | --  similar to *P. brasili*, but smaller (C.et M., 1919) | **ellipsoid to pear-shaped, circular in cross section** |
| **Trophozoite: length x width, µm** | 50 x 12 (von K. 1848); young 4-12 x 2-7; gamont 100 x 35 (C. & M. 1914) | young 7.5 x 3, gamont 180 х 36 (M. & R. 1931) | **30-65 х 10.5-22** | about 200 (C. & M. 1914a, 1919) | 31–385 x 10–76 | 30-60 x 20 | young 25 x 12.5 (F. 1936); 100-180 x 30-40 (M. & R. 1931); 90–297 x 34–135 (R. et al. 2018) | -- | -- | **28–288 x 14–50** |
| **Number of crests; apical filaments in the crest tips** | -- | -- | **2-6 crests/μm; --** | -- | up to 150, 3–5 crests/μm; -- | -- | up to 190, 3–5 crests/μm (R. et al. 2018); -- | -- | -- | **about 5 crests/ μm; 10-12 apical filaments** |
| **Attachment apparatus (epimerite or mucron by different authors)** | conical epimerite with prongs (sometimes branched) (C. & M. 1914) embedded into host cell; epimerite with 7-9 (usually 8) bifurcated prongs (R. 1932) | knob-shaped epimerite “extracellularly” attached to the host cells, with apical 2 large, diverging, claw-like processes, which are flattened towards their tips and merely applied to the surface of the epithelium, and a basal circlet of 14-16 minute teeth (M. & R. 1931) | **knob-shaped epimerite “extracellularly” attached to the host cell, with a collar at the base** | epimerite = *P. spionis*, but less developed, prongs smaller (C. & M. 1914a) | knob-like mucron, sometimes with up to 10 prongs at its base | epimerite of short cylinder shape, spreading out into 8 prongs firmly embedded in the host gut epithelium; clear zone of cytoplasm behind the epimerite | epimerite of truncated cone-shaped with a collar at its base (L. 1893); epimerite with a ring of prongs anterior to a collar (F. 1936); knob-like, 22-24 slender prongs anterior of the collar (M. & R. 1931); mucron flat (fractured?) or rounded protrusion (R. et al. 2018) | -- | -- | **domed-shaped epimerite, with a collar at the base, whole epimerite embedded in the host cell except the collar located “extracellularly”** |
| **Posterior end** | -- | -- | **rounded** | -- | rounded | tapering, pointed | rounded (R. et al. 2018) | -- | -- | **rounded** |
| **Nucleus shape and size (µm), number of nucleoli** | spherical, 1 nucleolus (M. 1891) | 10.5 х 7.5, two nucleoli (M. & R. 1931) | **--** | spherical, 1 nucleolus (fig. C1-3, E1 in C. & M. 1919) | spherical, 5–26 | spherical | spherical, x 15, 1 or more nucleoli (M. & R. 1931); spherical, x 14 – 29 (R. et al. 2018) | --  spherical, 1 nucleolus (Fig. 6-7, de F. et al. 1918) | -- | **spherical-oval, 9.5 – 19.0 x 9.5 – 17.0, with 1 large or 2-4 small nucleoli** |
| **Nucleus position and orientation** | in the middle or closer to anterior end (M. 1891) | in the middle (M. & R. 1931) | **in the widest cell part** | in the middle (fig. C1-3, E1 in C. & M. 1919) | towards the ends, in the largest cell part | -- | in the middle (M. & R. 1931; R. et al. 2018) | --  in the middle (Fig. 6-7, de F. et al. 1918) | -- | **in the widest cell part, longitudinally, closer to anterior or posterior ends** |
| **Nucleolus shape, size and position in nucleus** | -- | large and small nucleoli (M. & R. 1931) | **--** | spherical (fig. C1-3, E1 in C. & M. 1919) | -- | 1 large nucleolus | periphery and medulla well-defined (M. & R. 1931; F. 1936) | -- | -- | **spherical, x 3.7-8.5, x 2.7-5.9, in different nucleus parts** |
| **Syzygy** | -- | -- | **--** | -- | -- | -- | -- | -- | -- | **--** |
| **Motility of trophozoites** | contraction, gliding (von K. 1845, 1848); | quite immobile (?) (M. & R. 1931) | **gliding** | gliding (C & M. 1919) | gliding | gliding | -- | -- | -- | **gliding** |
| **Gametocyst** | -- | -- | **--** | -- | -- | -- | 98-105 x 90-100 (F. 1936) | -- | -- | **--** |
| **DNA sequences** | -- | *--* | ***--*** | *--* | -- | -- | *--* | *--* | *--* | **rDNA operon** |
| **Occurrence of archigregarines in the host (from different references)** | *Selenidium* *spionis* (Kolliker, 1845) Ray, 1930 [*Selenidium intraepitheliale* Reichenow, 1932];  *Selenocystis foliata* (Ray, 1930) Dibb, 1938 | *Selenidium* *spionis* (Kolliker, 1845) Ray, 1930 [*Selenidium intraepitheliale* Reichenow, 1932];  *Selenocystis foliata* (Ray, 1930) Dibb, 1938 | ***--*** | *Selenidium martinensis* Levine, 1971 (= *Selenidium* sp. Caullery et Mesnil, 1897) | -- | *Selenidium spinosis* Ganapati, 1946 | *Selenidium axiferans* Fowell, 1936 | *Selenidium cruzi* Faria, Cunha et Fonseca, 1917 | *--* | ***Selenidium pygospionis* Paskerova et al., 2018** |
| **Infection by *Metchnikovella* microsporidians (from different references)** | -- | *Metchnikovella* sp., similar to *M. spionis* Caullery, Mesnil, 1897 (M. & R. 1931) | ***--*** | *M. spionis* Caullery et Mesnil, 1897;  *M. brasili* Caullery et Mesnil, 1919 | -- | *Metchnikovella* sp., infected gregarines are deformed | *M. caulleryi* Mackinnon, Ray, 1931 | *--* | *M. incurvata* Caullery et Mesnil, 1914;  *M. oviformis* Caullery et Mesnil, 1914 | ***M. incurvata* Caullery et Mesnil, 1914;**  ***M. spiralis* Sokolova et al., 2014** |
| **Characteristic features (by different authors)** | epimerite embedded into the host cell. | epimerite extracellularly attached (clinging) to the host cells; infected host cells rarely hypertrophied | **--** | -- | -- | resembles *Polyrhabdina spionis*, but smaller, prongs not bifurcated | inhabit two polychaete hosts, or similar species?  affected host cells thickened (M. & R. 1931); epimerite sunk in the cell depression (M. & R. 1931; F. 1936); epimerite may be caducous (L. 1893) | -- | -- | **epimerite with collar, without prongs** |
| **References** | Caullery and Mesnil 1914a; von Kölliker 1845, 1848; Mingazzini 1891; Reichenow 1932 | Mackinnon and Ray 1931; Reichenow 1932 | **this paper** | Caullery and Mesnil 1897a,b, 1914a, 1919 | Rueckert et al. 2018 | Ganapati 1946 | Caullery and Mesnil 1914a; Kamm 1922; Makkinnon and Ray 1931; Fowell 1936; Rueckert et al. 2018 | de Faria et al. 1918; | Caullery and Mesnil 1914a; 1914b; 1919 | **this paper** |

Abbreviations: ‘—‘, no data; ‘?’, contradictory or vague description; ‘*’, species examined by electron scanning microscopy; ‘**’, species examined by electron scanning and transmission microscopy. The validation of the scientific names was conducted in the World Register of Marine Species (WoRMS).

References:

Caullery, M., Mesnil, F. 1914a. Sur l'existence de grégarines dicystidées chez les annélides polychètes. C. R. Soc. Biol. 77, 516-520.

Caullery, M., Mesnil, F. 1914b. Sur les Metchnikovellidae et autres protistes parasites des grégarines d'annélides. C. R. Soc. Biol. 77, 527-532.

Caullery, M., Mesnil, F. 1919. Metschnikovellidae et autres protistes parasites des Gregarines d' Annelides. Ann. Inst. Pasteur 33(4), 209-240.

Caullery, M., Mesnil, F.1897a. Sur trois Sporozoaires parasites de la *Capitella capitata* O.Fab. C. R. Soc. Biol. 49, 1005-1008.

Caullery, M., Mesnil, F.1897a. Sur un type nouveau (*Metchnikovella* n.g.) d'organismes parasites des grégarines. C. R. Soc. Biol. 49, 960-962.

de Faria, G., de Cunha, M., da Fonseca, O.R. 1918. Protozoarios parazitos de *“Polydora socialis*”. Mem. Inst. Osw-Cruz. 10, 17-19.

Fowell, R.R. 1936. Observations on the Sporozoa inhabiting the gut of the polychaete worm *Polydora flava* Claparède. Parasitology. 28, 414-430.

Ganapati, P.N. 1946. Notes on some gregarines from polychaetes of the Madras coast. Proc. Ind. Acad. Sci. Sect.B. 23(5), 228-248.

Kamm Watson, M. 1922. Studies on gregarinies II. Synopsis of the polycystid gregarines of the world, excluding those from the Myriapoda, Orthoptera, and Coleoptera. Illinois Biological Monographs. VII(1), 1-103.

Mackinnon, D.L., Ray, H.N. 1931. Observations on dicystid gregarines from marine worms. Quarterly Journal of Microscopical Science 74: 439-466

Mingazzini, P. 1891. Gregarine monocistidee, nuove o poco conosiute, del Golfo di Napoli. Atti Reale Accad. Lincei. Rendiconti. 7, 229-235.

Reichenow, E. 1932. Sporozoa. In: Grimpe, G., Wagler, E. (eds). Die Tierwelt der Nord und Ostsee. Leipzig. Lief 21 (Teil II), 1–88.

Rueckert, S., Glasinovich, N., Diez, M.E., Cremonte, F., Vázquez, N. 2018. Morphology and molecular systematic of marine gregarines (Apicomplexa) from Southwestern Atlantic spionid polychaetes. J. Invertebr. Pathol. 159, 49-60.

von Kölliker, A. 1845. Die Lehre von der thierischen Zelle und den einfacheren thierischen Formelementen, nach den neuesten Fortschritten dargestellt. Zeitschrift für wissenschaftliche Botanik. 1(2), 46-102.

von Kölliker, A. 1848. Beiträge zur Kenntniss niederer Thiere. Zeitschrift für wissenschaftliche Zoologie. 1, 1-37.
